# The Landscape of Coronavirus Disease 2019 (COVID-19) and Integrated Analysis SARS-CoV-2 Receptors and Potential Inhibitors in Lung Adenocarcinoma Patients

**DOI:** 10.3389/fcell.2020.577032

**Published:** 2020-10-23

**Authors:** Bufu Tang, Jinyu Zhu, Ying Cong, Weibin Yang, Chunli Kong, Weiyue Chen, Yajie Wang, Yong Zeng, Jiansong Ji

**Affiliations:** ^1^Key Laboratory of Imaging Diagnosis and Minimally Invasive Intervention Research, Lishui Hospital, School of Medicine, Zhejiang University, Lishui, China; ^2^Department of Radiology, Second Affiliated Hospital, School of Medicine, Zhejiang University, Hangzhou, China; ^3^Department of Microbiology, Center for Disease Control and Prevention, Lishui, China; ^4^Department of Radiology, The Fifth Affiliated Hospital of Wenzhou Medical University, Lishui, China; ^5^Qingtian County Center for Disease Control and Prevention, Lishui, China

**Keywords:** coronavirus disease 2019 (COVID-19), severe acute respiratory syndrome coronavirus 2 (SARS-CoV-2), lung adenocarcinoma (LUAD), immune infiltration, inflammation

## Abstract

The outbreak of novel coronavirus disease 2019 (COVID-19) has become the largest health threat worldwide, with more than 34.40 million positive cases and over 1.02 million deaths confirmed. In this study, we confirmed that significantly differentially expressed genes in COVID-19 patients were mainly involved in the regulation of immune and inflammation-related signaling pathways. It is worth noting that many infected COVID-19 patients have malignant tumors, and their prognosis is poor. To explore the susceptibility factors of cancer patients, we assessed the expression of ACE2, TMPRSS2, and the endocytic regulator AAK1 in lung adenocarcinoma (LUAD) patients and explored their effects on immune infiltration. We found that the expression of ACE2 and TMPRSS2 in LUAD patients was significantly increased, which may explain why LUAD patients are susceptible to SARS-CoV-2, and the patients with high-expression genes presented increased infiltration of immune cells such as B cells and CD4 T cells. In addition, we also identified miR-432-5p as a potential targeted molecule and bexarotene as a potential targeted drug of the three genes through bioinformatic analysis and further verified the anti-inflammatory effect of bexarotene, providing new ideas for the treatment of COVID-19.

## Introduction

The outbreak of coronavirus disease 2019 (COVID-19) caused by severe acute respiratory syndrome coronavirus 2 (SARS-CoV-2) began in Wuhan, China, in December 2019 (Gorbalenya et al., 2020) and then spread rapidly around the world (Berry et al., 2020; Vaid et al., 2020; Wang et al., 2020). As of Oct 2nd, 2020, the World Health Organization (WHO) has reported more than 34.40 million confirmed positive cases and over 1.02 million deaths^1^. COVID-19 is a potentially fatal disease that has aroused public health concerns worldwide, and its high morbidity and mortality pose a major challenge as a public health problem for China and many other countries.

The severity of COVID-19 can range from mild to moderate with infection symptoms such as fever, cough, and fatigue to severe and fatal with symptoms characterized by respiratory dysfunction and/or multiple organ failure (Huang et al., 2020; Rothan and Byrareddy, 2020). Currently, the identification of the progression of COVID-19 mainly depends on clinical manifestations. There are no established biomarkers that can effectively predict disease progression. The treatment strategy for COVID-19 patients mainly focuses on providing supportive care, such as oxygenation, ventilation, and infusion management, and there are no vaccines or recognized specific antiviral drug regimens for the treatment of critical patients (Kupferschmidt and Cohen, 2020; Tobaiqy et al., 2020).

Coronaviruses use their spike proteins to bind and enter target cells through specific receptors. Severe acute respiratory syndrome coronavirus (SARS-CoV) was confirmed to use angiotensin converting enzyme 2 (ACE2) as a receptor to enter cells, and SARS-CoV-2 and SARS-CoV have 79.5% homologous sequences. Both bioinformatics modeling and *in vitro* experiments demonstrated that ACE2 was recognized as the receptor for SARS-CoV-2 entry into target cells, and the cellular protease TMPRSS2 for SARS-CoV-2 spike protein priming is also essential for target cell entry and spread (Hoffmann et al., 2020; Yan et al., 2020; Zhou et al., 2020). It is worth noting that cancer patients are more likely to be infected with COVID-19 than patients without cancer (Longbottom et al., 2016; Liang et al., 2020). ACE2 is also aberrantly expressed in many tumors, such as LUAD (Chai et al., 2020). SARS-CoV-2 enters target cells through ACE2-mediated endocytosis, and AP2-associated protein kinase 1 (AAK1) is one of the known regulators of endocytosis (Richardson et al., 2020). The destruction of AAK1 may interrupt the spread of the virus to cells and the intracellular assembly of virus particles (Lu et al., 2020). Considering the crucial role of ACE2, TMPRSS2, and AAK1 in the SARS-CoV-2 invasion of target cells and intracellular transmission, performing an analysis of the expression and distribution characteristics and related biological processes of ACE2, TMPRSS2, and AAK1 in cancer patients can help us better understand the pathogenesis of their susceptibility to COVID-19 and better explore potential novel treatment strategies.

In this study, we first performed enrichment pathway analysis and protein-protein interaction (PPI) network analysis on genes with significantly different expression characteristics between COVID-19 patients and normal subjects by analyzing the GSE147507 dataset in order to explore the genes and their functions that change significantly in the progression of COVID-19. Then, we focused on the expression characteristics and functions of SARS-CoV-2 Receptors including ACE2, TMPRSS2, and AAK1 in LUAD as well as their relationship with the level of immune infiltration and explored the potential therapeutic targets and drugs for ACE2, TMPRSS2, and AAK1, then further indicated the anti-inflammatory effect of SARS-CoV-2 Receptors inhibitor bexarotene, providing promising ideas for the treatment of COVID-19.

## Materials and Methods

### Acquisition of Differentially Expressed Genes (DEGs) Between Healthy Subjects and COVID-19 Patients

The mRNA sequencing data of COVID-19 patients were obtained from the GSE147507 dataset (containing 2 normal samples and 2 COVID-19 patient samples) in the Gene Expression Omnibus (GEO) database (Blanco-Melo et al., 2020). Differential expression analysis is performed through the R package “edgeR.” Before performing the differential expression analysis, the variance of gene expression will be estimated; the degree of internal gene expression difference is estimated to see whether the difference in gene expression between groups is greater than the internal difference. If it is, the gene is defined as a differentially expressed gene. Then we use the package “edgeR” to standardize and the standardization method is as follows: In edgeR, the TMM method computes normalization factors that represent sample-specific biases. These factors are multiplied by the library size to yield the effective library size, i.e., the library size that we would have gotten if those biases were not present. The effective library sizes can then be used for various normalization purposes, most frequently as offsets in generalized linear models. We identified the obtained false discovery rate (FDR) as the adjusted *P*-value. The DEGs between COVID-19 patients and normal subjects were determined under the condition of absolute log2-fold change (FC) > 1 and the adjusted *P*-value < 0.05.

### Functional Enrichment Analysis

Gene Ontology (GO) analysis was performed in The Database for Annotation, Visualization and Integrated Discovery (DAVID)^2^. Kyoto Encyclopedia of Genes and Genomes (KEGG) pathway enrichment analysis of the DEGs was performed using ClueGO and CluePedia in Cytoscape software (version 3.5.1) (Shannon et al., 2003). *P* < 0.05 was considered to represent statistical significance.

### Protein-Protein Interaction Network Analysis

The BioGRID database^3^ was used to perform PPI network analysis on the proteins encoded by the identified DEGs (Stark et al., 2006). The PPI network was constructed in an online gene function analysis website- Cytoscape software^4^, and the key PPI network modules were selected by the Molecular Complex Detection (MCODE) plugin (Bader and Hogue, 2003).

### Estimation of the Infiltration of Multiple Types of Immune Cells

Cell-type Identification By Estimating Relative Subsets Of RNA Transcripts (CIBERSORT) analysis was applied to analyze the absolute abundance of 21 immune cells in heterogeneous tissues to evaluate the correlation between the level of immune cell infiltration and expression of ACE2, TMPRSS2, and AAK1 (Gentles et al., 2015). The R package “CIBERSORT” was used to convert the mRNA data of non-tumor cells into the tumor microenvironment infiltration level. For each sample, the sum of all estimated immune cell type scores is equal to 1. And Tumor Immune Estimation Resource (TIMER)^5^ (Li et al., 2017) was also used for interactively explore the associations between immune infiltrates and gene expression.

### Weighted Gene Co-expression Network Analysis (WGCNA)

The DEGs between LUAD patient samples and normal samples in the TCGA database were used to construct a gene co-expression network with the R package “WGCNA” (Langfelder and Horvath, 2008). First, the transcription data of the DEGs were entered to establish an expression matrix. The best “soft thresholding power” β was graphically determined to ensure the scale-free distribution network, and a co-expression matrix was constructed using the expression matrix and the β value. Using the DynamicTreeCut algorithm, genes with similar expression levels were classified into the same gene module, resulting in co-expression modules. The module Eigengenes function of the WGCNA R package was used to calculate the differences of the module eigengenes (ME) and the relevance of the modules to ACE2, TMPRSS, and AAK1. A heatmap was used to visualize the relevance of each module. Pearson’s coefficient was calculated to assess the correlations between the genes in the modules and ACE2, TMPRSS, and AAK1.

### Determination of the Co-regulated miRNA Factor for ACE2, TMPRSS, and AAK1

miRNAs targeted ACE2, TMPRSS2, and AAK1 were predicted in miRWalk^6^, miRanda^7^, RNA22^8^, Targetscan^9^, and RNAhybrid^10^ with different algorithms. The databases to predict targeted miRNA with different algorithms predict a set of miRNA IDs rather than specific regulated sequences. miRNAs which are repeatedly predicted in at least 2 databases are identified as the targeted miRNAs of the genes. Then we overlapped the IDs of the three sets of miRNAs predicted by the three genes to obtain the IDs of the co-regulated miRNAs of the three genes, and subsequently we used the RNAhybrid^10^ database to determine the specific sequences of co-regulated genes regulating these three genes.

### Exploration of the Potential Targeted Drug for ACE2, TMPRSS2, and AAK1

Potential targeted drugs for ACE2, TMPRSS2, and AAK1 were explored in the GDSC database^11^. We selected drugs whose response sensitivity is positively correlated with gene expression levels as the targeted drugs for the genes. And the targeted drugs for ACE2, TMPRSS2, and AAK1 were gathered to determine the potential targeted drug that can co-regulate these three genes.

## Results

### Characterization of DEGs Between Healthy Subjects and COVID-19 Patients

**FIGURE 1:**
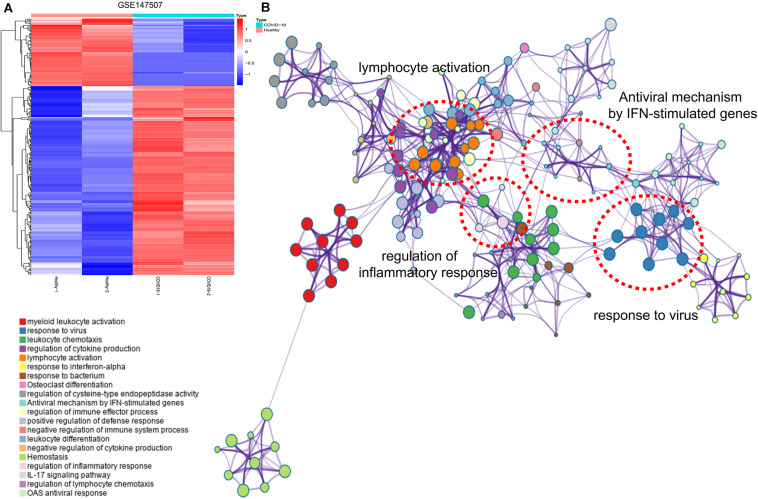
Hierarchical clustering analysis and KEGG analysis of DEGs. **(A)** Hierarchical clustering analysis showed the expression of DEGs. Each row represents a single gene and each column represents a sample. **(B)** KEGG analysis presented the main enrichment pathways of DEGs.

The expression profile of the DEGs between healthy subjects and COVID-19 patients is shown in Figure 1A. Hierarchical clustering analysis was adopted to categorize genes with similar expression profiles into two groups. Through KEGG analysis of these DEGs, we found that the genes are mainly enriched in inflammation and immune-related signaling pathways, such as “lymphocyte activation,” “response to virus,” “antiviral mechanism by IFN-stimulated genes,” “regulation of immune effector process,” and “regulation of inflammatory response” (Figure 1B).

### Protein-Protein Interaction Network Analysis of DEGs

**FIGURE 2:**
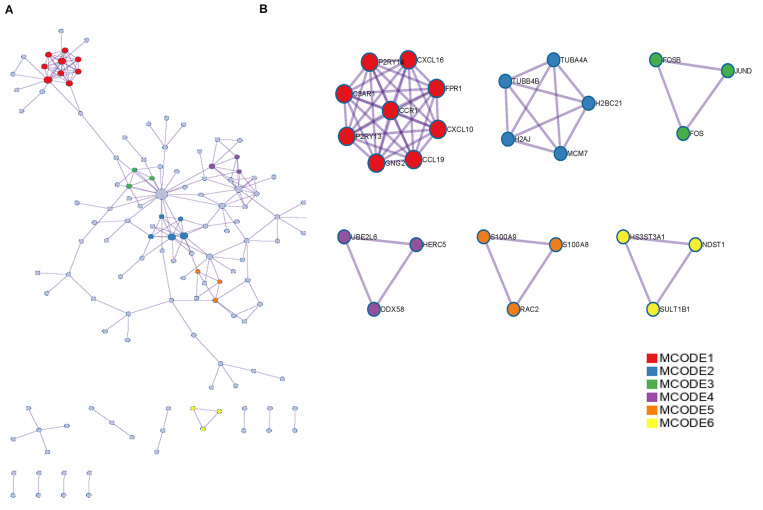
PPI network and MCODE components were associated with DEGs. **(A)** PPI network of proteins encoded by DEGs. **(B)** The essential modules identified by MCODE from the PPI network. Level >6 is set as the cutoff criterion.

We performed PPI network analysis on the proteins encoded by the identified DEGs in the BioGRID database and constructed the PPI network in the Metascape website (Figure 2A; Zhou et al., 2019). The MCODE plugin in Metascape was used to determine important modules and related hub genes in the constructed PPI network, and chemokines such as CCR1, CCL19, CXCL10, and CXCL16 were identified to present the most obvious changes in COVID-19 disease progression (Figure 2B).

### The Expression Profiles of ACE2, AAK1, and TMPRSS2 in LUAD Patients and COVID-19

**FIGURE 3:**
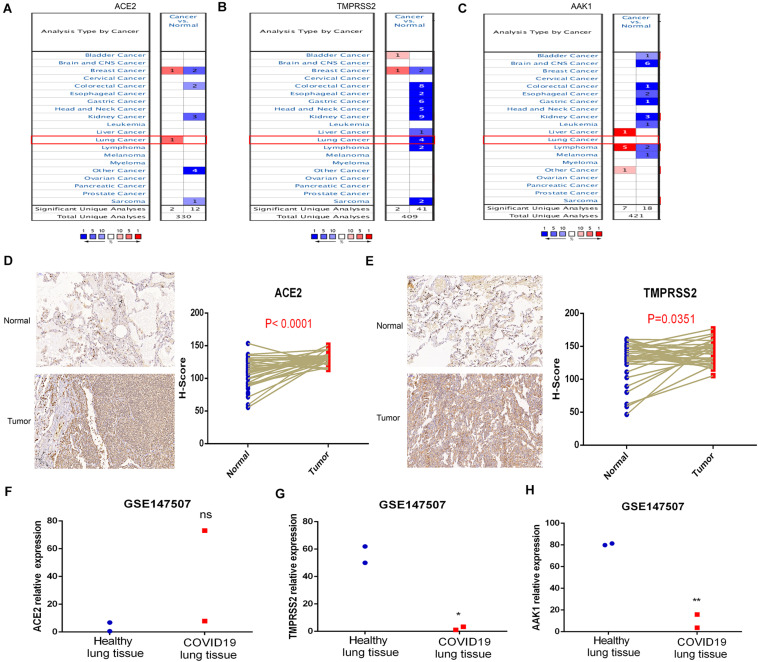
Expression characteristics of ACE2, AAK1, and TMPRSS2 in LUAD patients and COVID-19. **(A–C)** Differences in expression of ACE2 **(A)**, TMPRSS2 **(B)**, and AAK1 **(C)** between LUAD patients and normal subjects. **(D–E)** IHC staining showed expression patterns of ACE2 **(D)** and TMPRSS2 **(E)** in LUAD patients and normal samples. **(F–H)** Expression levels of ACE2 **(F)**, TMPRSS2 **(G)**, and AAK1 **(H)** in COVID-19 patients compared to normal subjects.

The expression characteristics of SARS-CoV-2 receptors including ACE2, AAK1 and TMPRSS2 between LUAD patients and normal subjects were analyzed in the Oncomine database. ACE2 and TMPRSS2 exhibited significant overexpression in LUAD patients (Figures 3A,B), while there was no significant difference in the expression level of AAK1 between LUAD patients and normal subjects (Figure 3C). Then, we evaluated the expression of ACE2 and TMPRSS2 in lung cancer patients through IHC staining, and markedly upregulated expression of the two genes was presented in lung cancer patients compared with normal samples (Figures 3D,E). The finding that ACE2 and TMPRSS2 are highly expressed in patients with lung cancer may be part of the reason why LUAD patients are more susceptible to SARS-CoV-2 infection. The expression patterns of ACE2, AAK1, and TMPRSS2 were also evaluated in COVID-19 patients in GSE147507 to explore gene changes in the progression of the disease (Figures 3F–H). The results showed that compared with normal subjects, the expression levels of AAK1 and TMPRSS2 in patients who had been infected with SARS-CoV-2 were significantly down-regulated, suggesting that COVID-19 patients may consume lots of AAK1 and TMPRSS2 in the advanced stage of the disease.

### The Expression Levels of ACE2, TMPRSS2, and AAK1 Are Closely Related to the Infiltration of Immune Cells

**FIGURE 4:**
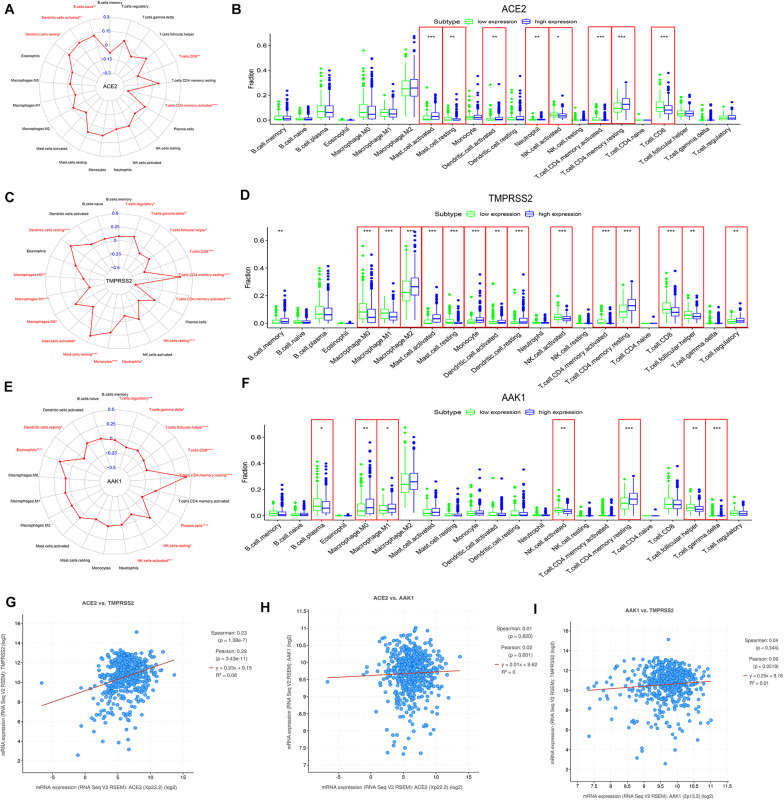
The correlation between expression patterns of ACE2, TMPRSS2, and AAK1 and the infiltration of immune cells in LUAD patients. **(A,C,E)** The expression levels of ACE2 **(A)**, TMPRSS2 **(C),** and AAK1 **(E)** are closely related to the infiltration characteristics of immune cells. **(B,D,F)** Differences in the infiltration of 21 immune cells between the high and low expression groups of ACE2 **(B)**, TMPRSS2 **(D)**, and AAK1 **(F)**. **(G–I)** Correlation between the expression characteristics of ACE2 and TMPRSS2 **(G)**, ACE2 and AAK1 **(H)**, and AAK1 and TMPRSS2 **(I)**. The “high” and “low” expression of ACE2 and TMPRSS2 are defined using the first and last quartile of the expression level.

The correlations between the infiltration of 21 immune cells and the expression levels of ACE2, TMPRSS2, and AAK1 performed through CIBERSORT analysis are presented in Figures 4A,C,E. The immune cells and their infiltration significantly affected by the expression levels of ACE2 and TMPRSS2 were relatively consistent; the infiltration levels of naive B cells, activated dendritic cells, CD4 memory T cells and CD4 regulatory cells were up-regulated in the ACE2 high-expression group and TMPRSS2 high-expression group (Figures 4B,D). The immune cell infiltration of patients with different AAK1 expression levels is presented in Figure 4F. The immune infiltration analysis results of TIMER2 were also relatively consistent with CIBERSORT analysis (Supplementary Figure 2A). The infiltration levels of B cells and CD4 T cells significantly increased in the groups with high expression of ACE2 and high expression of TMPRSS2 (Supplementary Figure 2B,C). And the AAK1 high-expression group also showed a high level of CD4 T cell infiltration (Supplementary Figure 2D). The cBioportal dataset^12^ (Cerami et al., 2012) showed ACE2 level was positively correlated with TMPRSS2 level (Figure 4G), the result maybe partially explained the consistence of immune cells infiltration. However, the correlation of other two genes was not significant difference (Figures 4H,I).

### WGCNA of DEGs Between LUAD Patients and Normal Subjects

**FIGURE 5:**
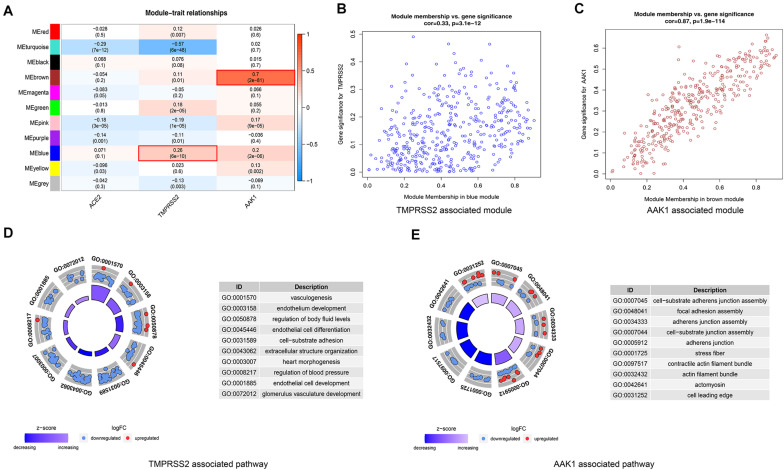
WGCNA of DEGs between LUAD patients and normal subjects for exploring potential mechanism and signaling pathway of ACE2, AAK1, and TMPRSS2. **(A)** Correlation of co-expression modules with ACE2, TMPRSS2 and AAK1. **(B)** Genes in the blue module are closely associated with TMPRSS2. **(C)** Genes in the brown module are highly correlated with AAK1. **(D)** GO analysis to explore signal pathways associated with TMPRSS2. **(E)** GO analysis to explore signal pathways associated with AAK1.

To further explore to potential mechanism and signaling pathway of SARS-CoV-2 receptors including ACE2, AAK1, and TMPRSS2 in LUAD patients. WGCNA analysis was used to identify the co-expressive modules. A total of 15415 mRNA-sequencing profiles (535 LUAD samples and 59 non-tumor samples) were obtained from The Cancer Genome Atlas (TCGA) (up to May 15, 2020). In addition, 4674 DEGs between LUAD patient samples and normal samples were selected with an absolute log2-fold change (FC) > 1 and an adjusted *P*-value < 0.05. WGCNA (Langfelder and Horvath, 2012) of the DEGs was performed to identify highly synergistically changing gene sets and to explore the associations between these gene sets and ACE2, TMPRSS2, and AAK1 (Supplementary Figure 1). We found that the “MEbrown” and “MEblue” modules were significantly related to both TMPRSS2 and AAK1, while none of the modules had a clear correlation with ACE2 (Figure 5A). Then, we explored the correlations of the genes in the modules with TMPRSS2 and AAK1. These genes were determined to be not only highly related to their corresponding modules but also significantly related to TMPRSS2 and AAK1 (Figures 5B,C). GO analysis was further performed on these genes in the two modules. The results indicated that the genes in the “MEbrown” module were enriched in biological functions and pathways such as “cell-substrate adherens junction assembly,” “focal adhesion assembly,” “adherens junction assembly,” “cell-substrate junction assembly” and “adherens junction,” and the genes in the “MEblue” module were enriched in “vasculogenesis,” “endothelium development,” “regulation of body fluid levels,” “endothelial cell differentiation” and “cell-substrate adhesion” (Figures 5D,E).

### miR-432-5p Is a Coregulated Factor of ACE2, TMPRSS2, and AAK1

**FIGURE 6:**
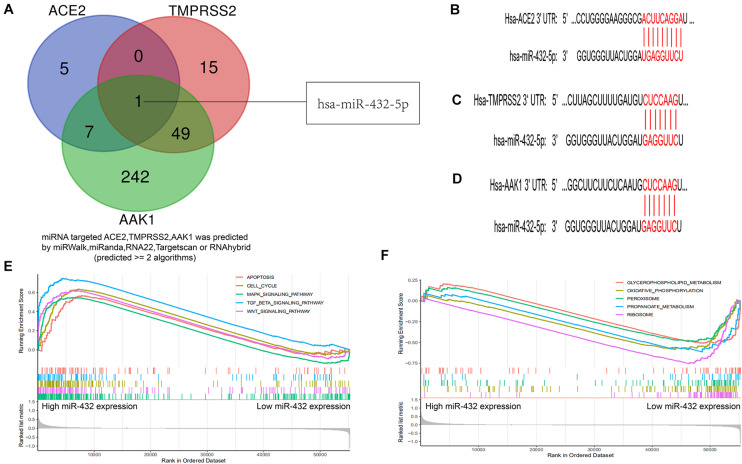
Bioinformatics identified miR-432-5p as a co-regulatory factor of ACE2, TMPRSS2, and AAK1. **(A)** Venn diagram exhibits miR-432-5p was a co-regulatory factor of ACE2, TMPRSS2, and AAK1. **(B–D)** Targeting sites of miR-432-5p and ACE2 **(B)**, TMPRSS2 **(C)**, and AAK1 **(D)**. **(E,F)** GSEA analysis presented top 5 positive regulatory pathways **(E)** and top 5 negatively regulatory pathways of miR-432-5p **(F)**.

MicroRNAs (miRNAs) are a class of small non-coding RNA molecules that regulate gene expression by targeting the mRNA 3′UTR (Fabian et al., 2010). To explore the potential inhibitor of SARS-CoV-2 receptors including ACE2, TMPRSS2, and AAK1, we performed bioinformatics analysis and used more than two different types of prediction algorithms to clarify that ACE2, TMPRSS2, and AAK1 were regulated by miR-432-5p (Figure 6A) (Supplementary Table 1). The targeting sites of miR-432-5p and the three genes are presented in Figures 6B–D. Then, we explored the signaling pathways with which miR-432-5p mainly interfered with physiological and pathological processes such as inflammation, oncogenesis and negative regulation via Gene Set Enrichment Analysis (GSEA) (Hänzelmann et al., 2013). The top 5 positively regulated pathways are shown in Figure 6E, and the top 5 negatively regulated pathways are shown in Figure 6F. The result suggested that miR-432-5p was potential agent of SARS-CoV-2 receptors and playing an important role in tumor progression in the LUAD patients.

### Bexarotene Is a Potential Target Drug of ACE2, TMPRSS2, and AAK1

**FIGURE 7:**
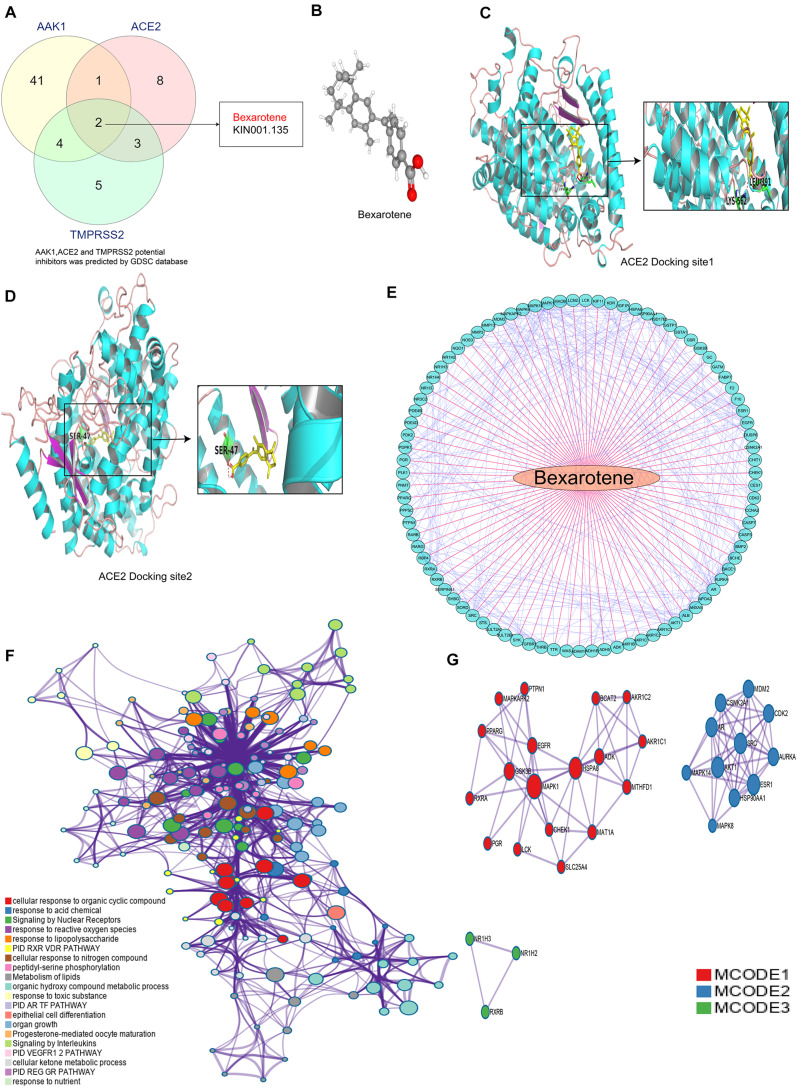
Bexarotene is a potential target drug of ACE2, TMPRSS2 and AAK1. **(A)** Venn diagram shows a Bexarotene is a potential targeted drug for all the three genes. **(B)** 3D structure of Bexarotene. **(C,D)** Possible docking site between Bexarotene and ACE2. **(E)** Correlation between genes regulated by Bexarotene. **(F)** The mainly signal pathways enriched by 87 genes regulated by bexarotene. **(G)** PPI network analysis of proteins encoded by the 87 genes.

In further exploring the clinical therapeutic value of targeting ACE2, TMPRSS2, and AAK1, we identified bexarotene as a potential targeted drug of all three genes in the GDSC database^13^ (Yang et al., 2013; Figure 7A and Supplementary Table 2–4). Bexarotene is a special retinoic acid X receptor (RXR) agonist, and RXRs play a key role in the proliferation, differentiation, apoptosis and angiogenesis of tumor cells through transcriptional regulation (Shen et al., 2018). The 3D structure of bexarotene is shown in Figure 7B. We used AutoDock software (version 3.6.1) to dock bexarotene with the three genes and found that bexarotene effectively docked with specific sites of ACE2 (Figures 7C,D). The docking analysis in AutoDock mainly showed hydrogen bonding between the gene and bexarotene. Bexarotene may bind to TMPRSS2 and AAK1 through ionic bonding, van der Waals forces or hydrophobic interactions, so AutoDock did not show the docking sites of bexarotene with these two genes. We further studied the specific mechanism of bexarotene in the Cancer Treatment Response Portal (CTRP) database^13^ and identified 87 genes that can be regulated by bexarotene. The correlation of these genes is shown in Figure 7E. GO analysis was performed to explore the signaling pathways in which the 87 genes were mainly enriched. The results demonstrated that these genes are mainly enriched in “cellular response to organic cyclic compound,” “response to acid chemical,” “signaling by nuclear receptors,” “response to reactive oxygen species,” and especially “response to lipopolysaccharide” signaling pathways (Figure 7F), indicating that bexarotene may participate in regulating inflammatory response. Figure 7G shows the core gene clusters within the 87 genes that have changed significantly during the development of LUAD.

### Bexarotene Treatment Inhibited LPS-Induced Inflammatory Response in the Macrophage

**FIGURE 8:**
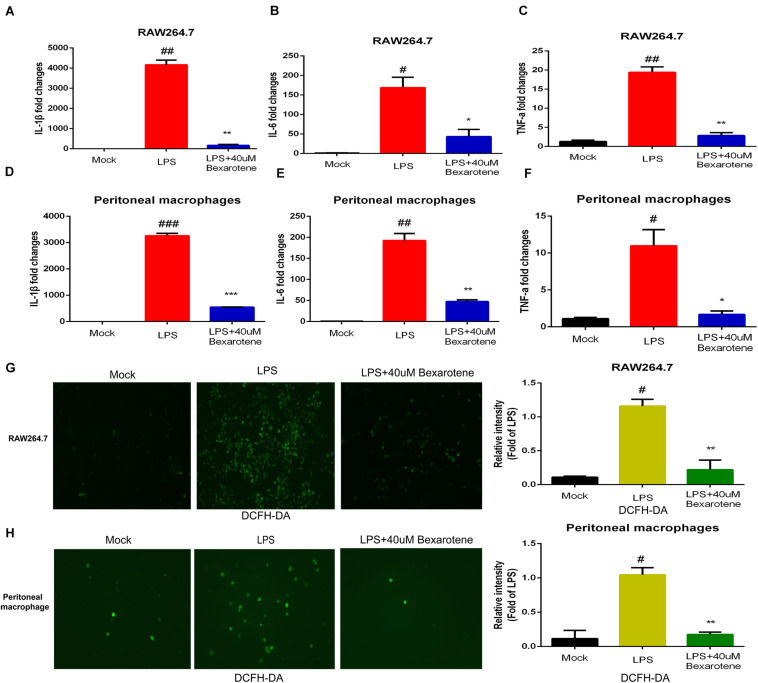
Bexarotene inhibits LPS-induced macrophage inflammation. **(A–C)** Bexarotene significantly inhibited mRNA levels of IL-1β **(A)**, IL-6 **(B)**, and TNF-α **(C)** in LPS-induced RAW264.7 macrophages. **(D–F)** Bexarotene showed significant inhibition on mRNA levels of IL-1β **(D)**, IL-6 **(E)**, and TNF-α **(F)** in LPS-induced peritoneal macrophage. **(G,H)** The ROS generation was obviously suppressed with bexarotene administration in the LPS-activated RAW264.7 macrophage **(G)** and peritoneal macrophage **(H)**.

To further explore the role of bexarotene in the inflammatory response, bexarotene was used to treated LPS-induced macrophage such as RAW264.7 and peritoneal macrophage. Then qPCR was used to measure the level of pro-inflammatory cytokines including IL-1β, IL-6 and TNF-a. The result showed the mRNA level of IL-1β, IL-6, and TNF-a was significantly inhibited in LPS-induced macrophage with bexarotene treatment both RAW264.7 macrophage and peritoneal macrophage (Figures 8A–F). In addition, DCFH-DA kit was used to detect reactive oxygen species (ROS) production in the LPS-induced macrophage. The result indicated the ROS generation was obviously suppressed with bexarotene administration in the LPS-activated macrophage including RAW264.7 macrophage and peritoneal macrophage (Figures 8G,H). The result suggested bexarotene treatment played an role in important in anti-inflammatory response, and the anti-inflammatory effect of bexarotene in the COVID-19 patients will also be further validated in our future research.

## Discussion

The global outbreak of COVID-19 caused by the new coronavirus SARS-CoV-2 has become the largest health threat in almost all countries, bringing an unprecedented burden on the global medical system (Sun et al., 2020). The natural and adaptive immunity of immune cells is activated after SARS-CoV-2 infects tissues to prevent further invasion of the virus. Most patients with COVID-19 only show mild to moderate infection symptoms, but some patients have a disorder between natural and adaptive immunity, which aggravates local tissue lesions and leads to excessive inflammation (Lee et al., 2020). In particular, the “cytokine storm” caused by the release of large amounts of cytokines and chemokines can lead to acute lung injury and respiratory distress syndrome, endangering the lives of patients (Chen et al., 2020; Xu et al., 2020).

In the current study, when we explored the signaling pathways of DEGs between COVID-19 and normal samples, we found that these DEGs were mainly enriched in “response to virus,” “lymphocyte activation,” “antiviral mechanism by IFN-stimulated genes” and “regulation of inflammatory response,” which were related to the physiological processes of inflammation and immunity, demonstrating that genes with altered expression in patients infected with SARS-CoV-2 were associated with the regulation of inflammation and immune response. Recently a study used a large number of bulk RNA sequences and single-cell sequencing data to show that the immune response of COVID-19 patients is driven by IFN and causes a large number of immune cell infiltration (Lee et al., 2020), which is also consistent with our results. And the subsequent PPI analysis results in our study also confirmed that chemokines related to inflammatory responses, such as CCR1, CCL19, and CXCL10, and the immune response-related gene FPR1 presented significant expression changes in the progression of COVID-19.

Previous studies confirmed that SARS-CoV-2 invades host cells through the ACE2 receptor (Rivellese and Prediletto, 2020), and ACE2 is expressed not only in the lung but also in the gastrointestinal tract, kidney, heart, blood vessels and other organs and tissues, causing SARS-CoV-2 to damage not only the lung but also the heart and kidneys, as well as physiological systems such as the immune system and blood system, causing death in patients due to multiple organ failure and acute respiratory distress syndrome (Bourgonje et al., 2020). TMPRSS2 has also been studied and demonstrated to cleave at the spike proteins S1/S2 and S2 of the virus to initiate the conformational change of the spike protein and promote the entrance of the virus into the host cell through membrane fusion or endocytosis (Stahlmann and Lode, 2020). The expression of ACE2 and TMPRSS is the key factor affecting the first step of SARS-CoV-2 infection. It is worth noting that previous studies revealed that many infected COVID-19 patients were patients with malignant tumors, suggesting that tumor patients are susceptible people who need important attention in the prevention and control of COVID-19 (Liang et al., 2020). In this study, we analyzed the expression characteristics of ACE2, TMPRSS and the endocytosis regulator AAK1 in LUAD patients and found that the expression levels of ACE2 and TMPRSS in LUAD patients were significantly higher than those in normal subjects, indicating that the high expression of these genes in LUAD patients may be one of the reasons they are more susceptible to SARS-CoV-2 infection. However, some studies found that ACE2 and TMPRSS2 will decrease during disease progression (Kong et al., 2020), which may be related to the massive invasion of viruses and the consumption of these receptors. And our analysis results also consistently showed that the TMPRSS level of COVID-19 patients is significantly lower than that of normal subjects.

In the subsequent analysis, the infiltration characteristics of immune cells in the tumor tissue of LUAD patients were revealed to be closely related to the expression levels of ACE2, TMPRSS, and AAK1. Patients with high expression of ACE2 and patients with high expression of TMPRSS had relatively identical infiltration changes of immune cells. Compared with patients with low expression of these genes, patients with high expression exhibited higher infiltration fractions of B cells, activated dendritic cells and CD4 T cells. CD4 T cells play a critical role in attenuating or suppressing overactive innate immune responses during viral infection to maintain self-tolerance and immune homeostasis (Shaw et al., 2013). The significantly increased infiltration of CD4 T cells and B cells in LUAD patients with high expression levels of ACE2 and TMPRSS2 may cause an imbalance in immune regulation, making patients more susceptible to SARS-CoV-2 invasion, and the inflammatory response after infection is aggravated. While in the advanced stage of COVID-19, the CD4 cells exhibit functional exhaustion and the absolute number of them decreased significantly (Qin et al., 2020; Tan et al., 2020), and the expression of ACE2 decreased as well.

The gene sets identified by the WGCNA of DEGs between LUAD patients and normal subjects with high correlations with ACE2, TMPRSS2, and AAK1 and highly synergistic changes had biological functions and signaling pathways mainly concentrated in “cell-substrate junction assembly,” “cell-substrate adhesion,” “vasculogenesis” and “endothelium development,” which were related to the development of tissue cells and cell-mediated immunity (Tozeren, 1990).

In further exploring potential current treatment options for ACE2, TMPRSS2 and AAK1, we identified miR-432-5p as a potential targeting molecule with docking sites and regulatory functions for all three genes. MiR-432-5p plays a key role in tumor progression by regulating gene expression at the posttranscriptional level. MiR-432-5p has been revealed to inhibit the progression of LUAD by inhibiting the cell cycle to suppress cell proliferation (Chen et al., 2016). Studies have also found that miR-432 can negatively regulate the production of MCP-1 and the migration of monocytes to inhibit the inflammatory response (Liu et al., 2017). The specific regulatory effect of miR-432-5p on the expression of ACE2, TMPRSS2, and AAK1 is not yet clear, and we will further explore the mechanism of miR-432-5p in future studies. In addition, pharmacological analysis suggested that bexarotene is a potential targeted drug of these three genes. Bexarotene is a synthetic high-affinity retinoid X receptor agonist that is currently used clinically for the treatment of T-cell lymphoma (Talpur et al., 2002). In preclinical mouse models, bexarotene has also been confirmed to inhibit the occurrence of lung tumors (Zhang et al., 2019). Some studies have demonstrated that bexarotene exerts anti-inflammatory effects by downregulating the expression of interleukin (IL)-6, IL-8, monocyte chemoattractant protein 1 (MCP-1) and high mobility group box-1 (Li et al., 2019). In addition, we found bexarotene treatment obviously inhibited LPS-activated inflammatory response and ROS generation in the macrophage. These results suggest bexarotene maybe regard as a promising therapeutic strategy for COVID-19. Next, the anti-inflammatory mechanism of bexarotene and its regulating effects on ACE2, TMPRSS2, and AAK1 will also be further explored and validated in our follow-up studies.

Inevitably, the small dataset of COVID-19 with only 2 patients and 2 normal subjects is indeed the limitation of our study, which may affect the credibility of the results. Regrettably, as of now, the GSE147507 that we analyzed in this study is the only publicly available database containing COVID-19 patient information. In the future, we will conduct more in-depth analysis after obtaining more information of COVID-19 patients. Besides, whether bexarotene plays a role in patients with COVID-19 requires a large amount of preclinical data support, which is also the work we need to further explore.

## Data Availability Statement

The original contributions presented in the study are included in the article/Supplementary Material, further inquiries can be directed to the corresponding author/s.

## Author Contributions

JJ conceived and designed the experiments and edited the manuscript. BT, JZ, and YC performed the experiments. YC, WY, and YW analyzed the data. CK, WC, and YZ contributed analysis tools. BT and JZ wrote the manuscript. All authors read and approved the final manuscript.

## Conflict of Interest

The authors declare that the research was conducted in the absence of any commercial or financial relationships that could be construed as a potential conflict of interest.

## Footnotes

1 https://covid19.who.int/

2 https://david.ncifcrf.gov

3 https://thebiogrid.org/

4 https://metascape.org/gp/index.html

5 http://cistrome.dfci.harvard.edu/TIMER/

6 http://mirwalk.umm.uni-heidelberg.de/

7 http://www.miranda.org/

8 https://cm.jefferson.edu/rna22/

9 http://www.targetscan.org/vert_72/

10 https://bibiserv.cebitec.uni-bielefeld.de/rnahybrid/

11 https://www.cancerrxgene.org/

12 http://www.cbioportal.org/

13 http://portals.broadinstitute.org/ctrp/

## Supplementary Material

Supplementary Figure 1 WGCNA for identifying co-expression modules. **(A)** Volcano Plot exhibited DEGs between LUAD patients and normal samples. **(B)** Identification of the optimal soft threshold. **(C–E)** Construction and visualization of co-expression modules.

Supplementary Figure 2 The infiltration of immune cells of LUAD patients with different expression characteristics of ACE2, TMPRSS2 and AAK1. **(A)** The correlation between expression of ACE2, TMPRSS2 and AAK1 and the infiltration of immune cells. **(B–D)** The infiltration of immune cells in the high and low expression groups of ACE2 **(B)**, TMPRSS2 **(C)**, and AAK1 **(D)**.

Supplementary Table 1 miRNA-target predicted by miRWalk, miRnda, RNA22, Targetscan, or RNAhybrid (predicted = 2 algorithms).

Supplementary Table 2 Sensitive drugs associated with ACE2 expression were predicted by GDSC database.

Supplementary Table 3 Sensitive drugs associated with TMPRSS2 expression were predicted by GDSC database.

Supplementary Table 4 Sensitive drugs associated with AAK1 expression were predicted by GDSC database.

## Abbreviations

ACE2: angiotensinconverting enzyme 2
COVID-19: Novel coronavirus disease 2019
CTRP database: Cancer Treatment Response Portal database
DEGs: differentially expressed genes
LUAD: lung adenocarcinoma
TCGA database: The Cancer Genome Atlas database
WGCNA: weighted gene coexpression network analysis
WHO: World Health Organization.
