# Supplementary figures and images for: The Landscape of Coronavirus Disease 2019 (COVID-19) and Integrated Analysis SARS-CoV-2 Receptors and Potential Inhibitors in Lung Adenocarcinoma Patients
Source: Front Cell Dev Biol. 2020 Oct 23;8:577032. doi: 10.3389/fcell.2020.577032 (PMC7644800; doi:10.3389/fcell.2020.577032)

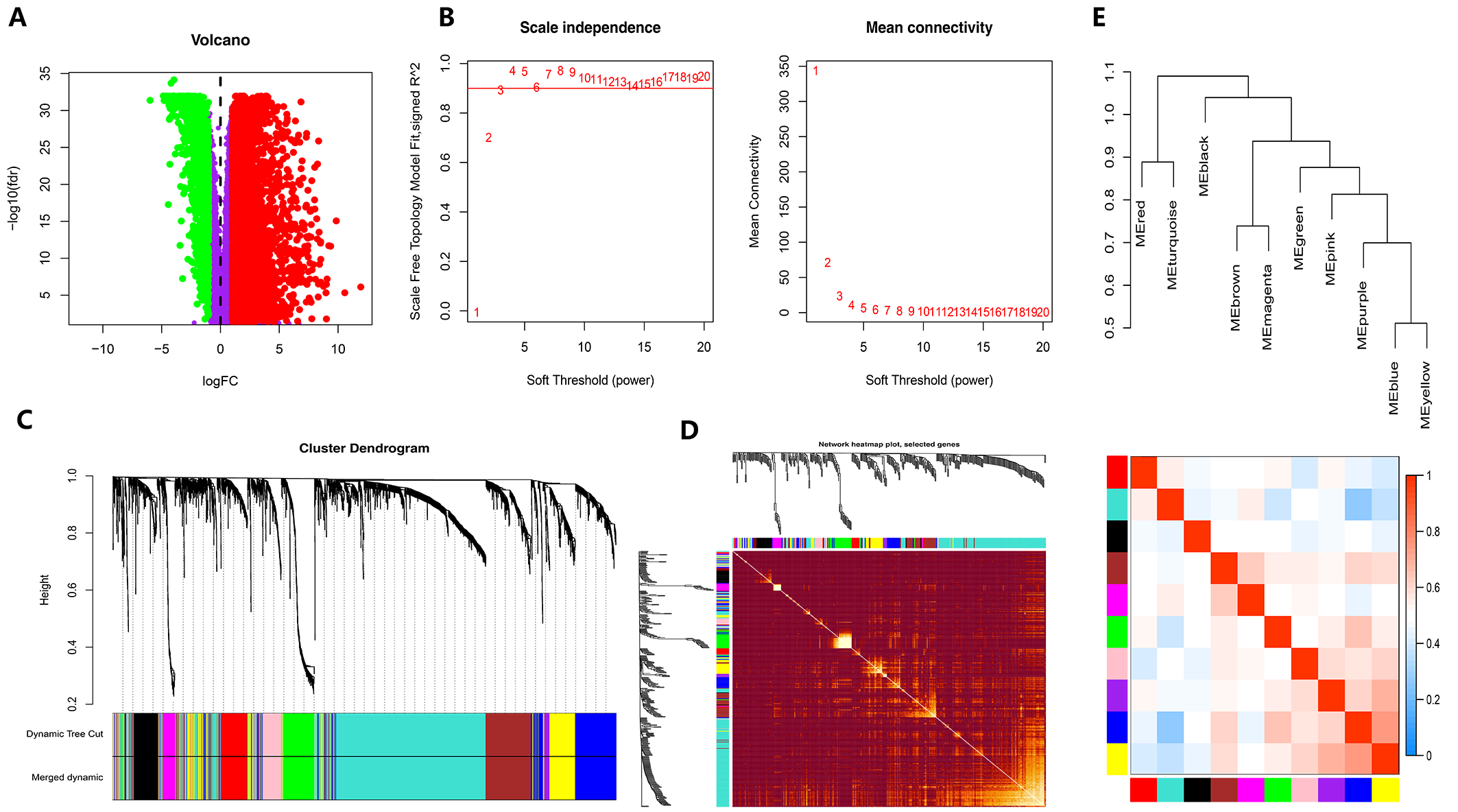

Supplement: Supplementary Figure 1 — WGCNA for identifying co-expression modules. (A) Volcano Plot exhibited DEGs between LUAD patients and normal samples. (B) Identification of the optimal soft threshold. (C–E) Construction and visualization of co-expression modules. [file Image_1.TIF]

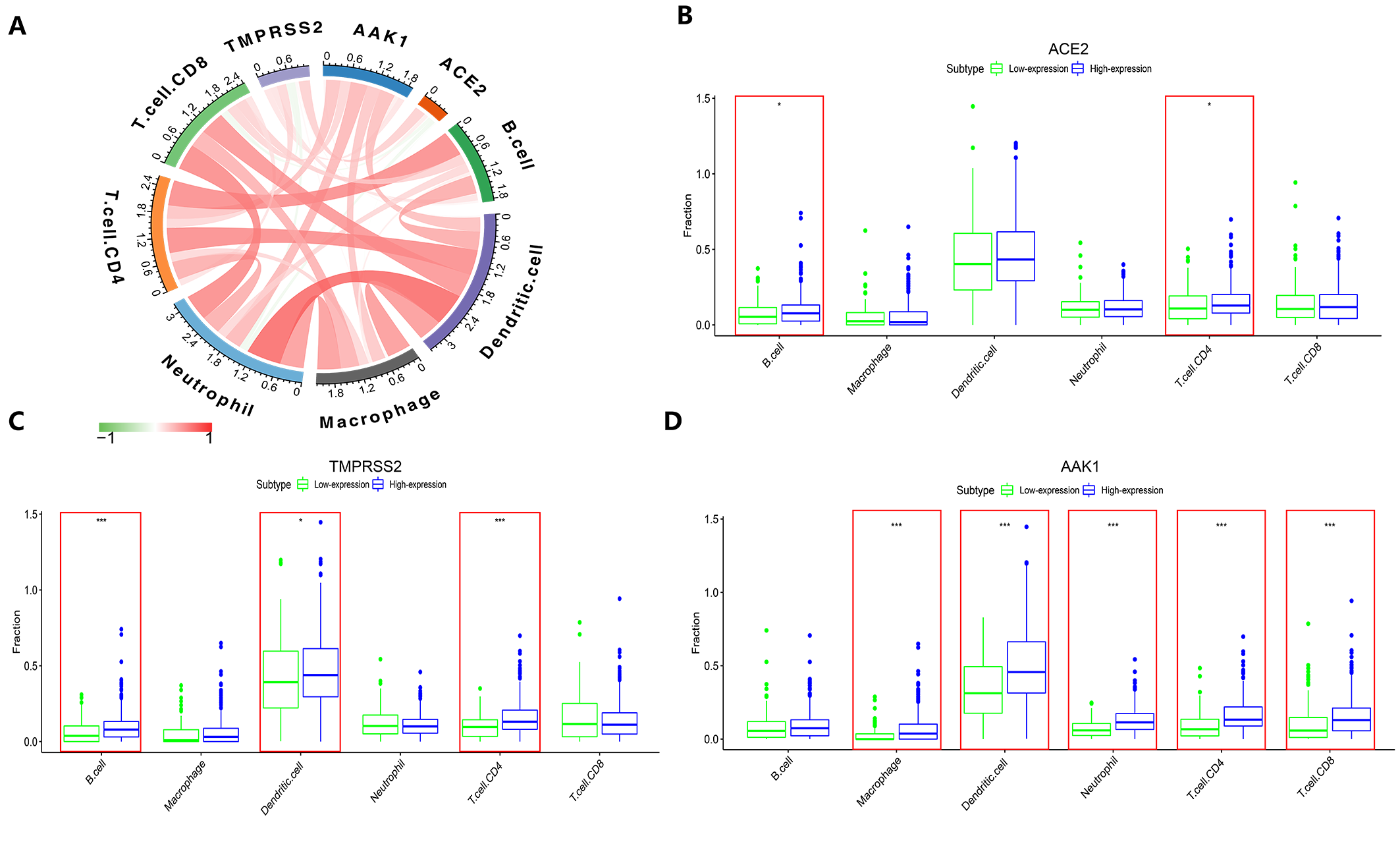

Supplement: Supplementary Figure 2 — The infiltration of immune cells of LUAD patients with different expression characteristics of ACE2, TMPRSS2 and AAK1. (A) The correlation between expression of ACE2, TMPRSS2 and AAK1 and the infiltration of immune cells. (B–D) The infiltration of immune cells in the high and low expression groups of ACE2 (B), TMPRSS2 (C), and AAK1 (D). [file Image_2.TIF]
